# Hand and Abrasive Flow Polished Tungsten Carbide Die: Optimization of Surface Roughness, Polishing Time and Comparative Analysis in Wire Drawing

**DOI:** 10.3390/ma15041287

**Published:** 2022-02-09

**Authors:** Raman Kumar, Sehijpal Singh, Vivek Aggarwal, Sunpreet Singh, Danil Yurievich Pimenov, Khaled Giasin, Krzysztof Nadolny

**Affiliations:** 1 Department of Mechanical and Production Engineering, Guru Nanak Dev Engineering College, Ludhiana 141006, India; sehijpalsingh@yahoo.in; 2 Department of Mechanical Engineering, IKGPTU Main Campus, Kapurthala 144603, India; dr.vivekaggarwal@ptu.ac.in; 3 Department of Mechanical Engineering, Chandigarh University, Ghauruan 140413, India; snprt.singh@gmail.com; 4 Department of Automated Mechanical Engineering, South Ural State University, Lenin Prosp. 76, 454080 Chelyabinsk, Russia; danil_u@rambler.ru; 5 School of Mechanical and Design Engineering, University of Portsmouth, Portsmouth PO1 3DJ, UK; khaled.giasin@port.ac.uk; 6 Department of Production Engineering, Koszalin University of Technology, Raclawicka 15-17, 75-620 Koszalin, Poland; krzysztof.nadolny@tu.koszalin.pl

**Keywords:** abrasive flow polishing, hand polishing, surface roughness, wire drawing die, tungsten carbide, TOPSIS, polishing time, abrasive flow machining

## Abstract

This research work highlights the benefits of abrasive flow polishing (AFP) applied to tungsten carbide dies compared with conventional hand polishing (HP). An indigenous experimental set-up for AFP was developed. The effect of prominent process parameters viz. extrusion pressure, number of cycles, and abrasive particle concentration on the final surface roughness, percentage improvement in surface roughness, and polishing time was investigated by Taguchi-designed experiments. The multi-objective optimization (MOO) was performed using the Taguchi-TOPSIS-Equal weight approach to find the respective optimized AFP parametric settings. A set of skilled operators performed the conventional HP of dies, and the best hand-polished (HPed) die was selected using the TOPSIS technique. The operational performance of the HPed dies and the abrasive flow polished (AFPed) dies were compared on the three-stage wire drawing operation. The results revealed that AFP’s surface resulted in a better-quality surface than hand polishing with a 27.06% improvement in surface roughness. Furthermore, AFP can reduce the dependency on costly and tricky-to-locate skilled operators, with a reasonable amount of time saving (about 87.05%). Overall, the study’s findings show that abrasive flow polishing of dies is fast and cost-effective.

## 1. Introduction

Polishing is a type of finishing operation performed after machining, and is essential to attain final surface quality and shape accuracy. It is generally performed on dies in metal forming and drawing operations to improve die life, quality of material processed, production time, and operation efficiency. However, inadequate polishing of the die leads to reduced die life and low product quality. In most industries, the majority of polishing operations are completed by the operators manually. The operator’s skill is responsible for the surface quality and shape correctness of the polished die. The skilled operators spend a lot time attaining the necessary roughness on the die surface. The die polishing operations consume almost 30% to 40% of the overall die manufacturing time. The skilled operators usually avoid polishing operations due to poor working environment owing to dust and noise pollution. It is recognized that the manual polished surface appears good, but this operation smears the metal, and the polished surface may contain folded metal instead of parent metal. After a few drawing cycles, the folded metal becomes weak, and gets pulled out by the wire drawing metal, thereby damaging the die [1,2].

Tungsten carbide is generally utilized in wire drawing dies to meet quality, uptime, and speed standards. However, die life shortens due to poor surface finish, leading to early failure of the die. The poor surface finish of the die also leads to die defects such as U-shaped cracks, pitting, wear rings, and vertical and horizontal cracks. In the drawing operation, the wire contacts the die hole repeatedly, loosening the tungsten carbide particles and forming a rough surface. The rough surface scrapes metal pieces of the steel wire’s surface, causing the lubrication to weaken and the wear rate to increase [3,4]. Lilly et al. [5] reported, while reviewing the automated finishing of dies, that out of the total time spent on die or mold manufacturing, 37% to 50% of time was wasted on polishing the dies. These operations are carried out by expert operators using conventional methods. The automation of this operation becomes difficult due to the high intensity of expertise and ability needed. Moreover, this situation arises due to a shortage of polishing parameter data and surface features, even though little research work has been reported in this field [6]. Therefore, to achieve better productivity and to resolve the probable scarcity of skilled operators, more than a few papers have reported on automated polishing.

The polishing on cold forging dies was completed with expert and non-expert operators, and their skill was measured by checking surface roughness, work time, and the variation from the designated thickness. The authors reported a disparity among expert and non-expert operators in terms of operating time and repetition accuracy. The operator needs several years to become an expert or to be skilled in polishing dies. Kito et al. [7] also investigated the polishing of cold-forged die with a skilled and non-skilled operator, and termed polishing as a removal operation. Tian et al. [8] performed automatic robotic polishing on NAK80 steel mold parts to diminish manual finishing costs and improve quality. The robot-assisted polishing of die and mold were investigated to enhance efficiency, reliability, and robustness [9,10].

Abrasive flow machining (AFM) was recognized as one of the promising technologies for fine finishing of hard and tough surfaces during the early seventies, but Kohut [11] was the first researcher who highlighted the application of AFM to polish extrusion dies, and reported that hand polishing leads to the folding of the metal. The AFM can remove the recast layer produced by EDM and achieve roughness equal to 0.05 μm [12]. The experimental work was conducted to comprehend the mechanism of material removal and the wear comportment treated by AFM and magnetically assisted AFM [13] The centrifugal force-assisted AFM was applied to study the effect of shape, rotational speed, extrusion pressure, number of operation cycles, and abrasive grit size. The input variables significantly affect material removal and surface roughness [14]. The AFM can finish complicated shapes and geometries and is ideal for polish operations because dies offer constraints to flowing media; thus, removing the prerequisite of a fixture [15]. Taguchi’s experiment methodology was applied to finish conventional machined cylindrical surfaces with AFM [16]. The AFM could easily remove EDM-damaged layers [17]. The AFM set-up was designed and fabricated to the micromachine hole of the nozzle [18]. A mechanism was developed to improve polishing effects on the workpiece surface by designing different passageways. This resulted in the uniform roughness of the circular holes [19]. The AFM process was utilized to enhance a cycloidal pump’s outer rotor’s surface feature and achieved 0.054 µm Ra of tooth top, 0.094 µm Ra of the tooth surface, and 0.185 µm Ra of tooth root along with 0.4 g removed material [20]. The surface quality results were achieved successfully by applying AFM to helical gear [21]. A polymer abrasive gel was developed for AFM to polish, and to trim the die of HSS. The achieved results were comparable with commercially available media with higher costs than the developed media [22]. The AFM was applied to finish the stainless-steel knee joint. The AFM operation reduced machining time by 76.56% compared with the ball-end tool utilized to finish the knee joint [23]. The AFM process achieved a surface roughness enhancement of 92.20%, while nano-finishing surgical stainless steel 316L tubes [24,25] optimized AFM parameters viz. abrasive concentration, extrusion pressure, and the number of cycles by the Taguchi method to improve surface finish. The chemical-AFM was designed to polish the interior surfaces of tubular IN625 parts and achieved enhanced texture and surface free form semi-welded particles [26]. The recent advancements in the AFM are described in the context of other media of lower prices, the expansion of tools and fixtures, and ecological matters [27]. Yunus and Alsoufic [28] optimized abrasive flow parameters while machining composite material previously cut with wire EDM. Bhardwaj et al. [29] highlighted that the AFM process has innovative modifications to develop a well-organized practice to achieve a better class product with a superior finish. The technical quality of the steel surface produced by laser and abrasive water jet cutting was evaluated [30]. An abrasive water jet cutting was used to assess the impact of a curve form cut out in a brittle material [31].

TOPSIS is a compensatory aggregation technique comparing a collection of choices by identifying the weight of every characteristic, normalizing and calculating the geometric distance from each choice to the ideal option, which is the best mark for each yardstick. TOPSIS allows trade-offs between criteria: i.e., another criterion’s promising outcomes can disallow one disappointing performance. This gives more authentic modelling than non-compensatory approaches that include or exclude hard cut-off choices [32,33]. The TOPSIS technique was utilized to attain optimal solution during ultrasonic-assisted electrochemical magnetic abrasive machining [34]. The TOPSIS method was used in conjunction with entropy weights to achieve optimum energy consumption, surface quality and production rate during the turning process [35]. TOPSIS was used for ordering the experiments the during EDM micro-drilling process [36]. The Taguchi–TOPSIS method has been utilized in electrical discharge machining to enhance machined surface quality and productivity [37]. TOPSIS, in conjunction with the Taguchi method, was successfully applied in machining operations for multi-objective optimization, and TOPSIS is also a well-known technique for ranking alternatives or experiments based on the compensatory aggregation technique [38].

The literature review reveals that abrasive flow machining is a well-established technology for fine finishing intricate profiles and geometries. However, the literature is scant to compare and validate the application of AFP to replace conventional die polishing methods, which are labor-intensive and time-consuming. AFP seems one of the possible methods that can be used for the polishing of dies, as it can promote the product’s inner surface quality and economize the labor cost. Hand polishing of carbide dies is performed frequently in industries, but the literature is silent with regard to applying abrasive flow machining to enhance surface roughness and machining time. Polishing a die with free-form surfaces is a time-consuming and onerous task requiring a high level of precision, and great accuracy is needed to keep the requisite size. Several employees have begun to shun polishing work because of the unpleasant working environment created by dust and noise. This method is incapable of achieving the fine polishing required for efficient wire drawing. In addition, the surface finish of the wire drawing die determines the quality of the wire and die life. The hand polishing operation cannot achieve the required surface finish, and it shortens the die life. Therefore, it is necessary to suggest and explore new and advanced methods to polish the die.

So, in the present study, the multi-objective optimization (MOO) of responses such as final surface roughness (F-Ra), percentage improvement of surface roughness (% age I-Ra), and total polishing time (TPT) was accomplished with the technique of the Taguchi-TOPSIS Equal-Weight. Furthermore, the TOPSIS method was also utilized to select the best hand-polished die, and the results were compared with abrasive flow polished die. The performance of AFPed and HPed dies in the context of bearing diameter and percentage reduction in surface roughness was also evaluated in a wire drawing operation that included abrasive flow and hand polishing.

## 2. Materials, Method, and Experimentation

**Figure 1:**
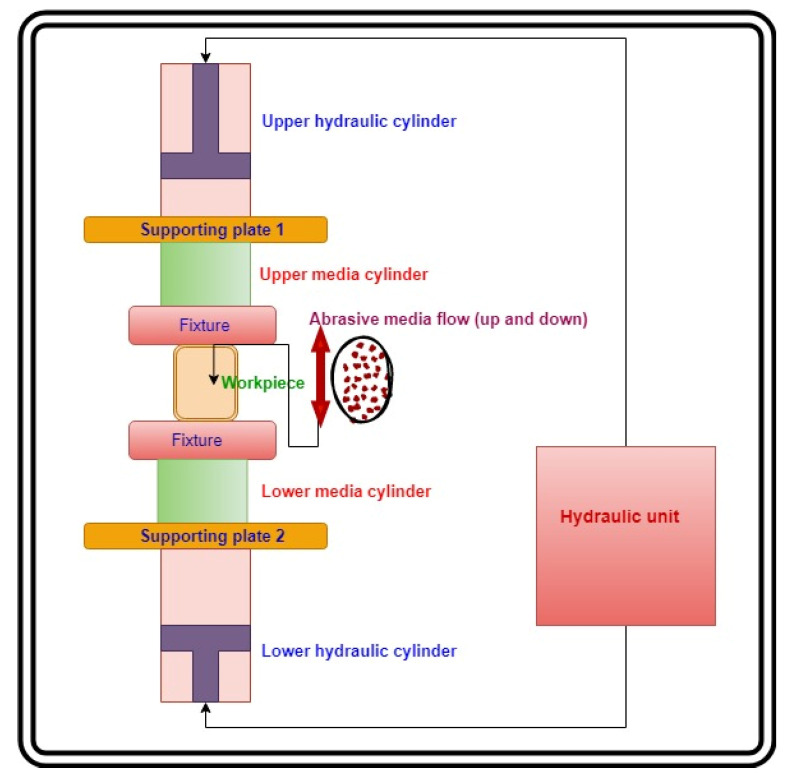
Abrasive flow machining set-up.

Tungsten carbide (WC) wire drawing dies were taken as workpieces in the present research. WC had a hardness of 8.5–9 on Moh’s scale at 22 GPa, a molar mass of 195.86 g/moL with the grey appearance of density 15.8 g/cm^3^, a melting point of 2870 °C, a boiling point of 6000 °C. It was also insoluble in water with a hexagonal crystal structure, according to the information provided by the supplier (Fine Spark, Pune, India). An indigenously developed AFP machine in the laboratory (Figure 1) was used to polish the WC dies. The abrasive media was prepared by mixing boron carbide abrasives with silicon-based polymer and hydrocarbon gel. Whereas the polymer provided necessary stiffness to the media, the gel facilitated the media’s smooth movement through the workpiece cavity.

### 2.1. Experimental Set-Up 1: Abrasive Flow Polishing

Figure 1 describes the working parts of the abrasive flow polishing machine. Figure 1 illustrates the functional features of the AFP machine set-up. It is prepared by arranging various hydraulic system components according to their functions. The lower media cylinder was packed with abrasive media while maintaining the piston and upper hydraulic cylinder at the apex point. The workpiece (die) was put in a fixture place. The fixture directed the media from the lower cylinder to the upper cylinder through the die. The powerpack or hydraulic unit generated hydraulic pressure in the die passage, as the die diameter was relatively smaller than the diameter of the media cylinders. Due to the constriction in media flow, the extrusion pressure was generated, as indicated on the pressure gauge. The movement of media from the lower cylinder to the upper cylinder and then back to the lower cylinder was termed as a cycle. A couple of cycles were needed to create a fine finish on the die passage.

The effects of three prominent parameters of AFP viz. extrusion pressure (Ep), number of cycles, and abrasive concentration (Ac) on the performance characteristics, such as surface roughness (Ra), percentage improvement of Ra, and polishing time, have been studied. The parameters were decided as per the trial of experiments and literature review [39]. The levels of variables are shown in Table 1. The experimental trials were conducted as per standard Taguchi L9 orthogonal array [40]. Experiments were designed and statistically analyzed with Minitab 16 software. The performance characteristics were recorded against various trials, and are shown in Table 2.

### 2.2. Experimental Set-Up 2: Hand Polishing

**Figure 2:**
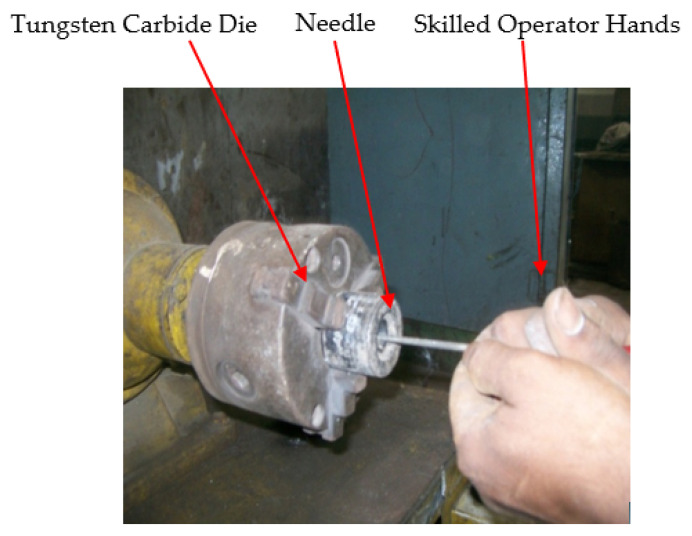
Hand polishing of die by the skilled operator.

The pictorial view of the special-purpose machine for the hand polishing (HP) of WC wire drawing dies is shown in Figure 2. It consists of a grinding machine, a three-jaw chuck is fastened in place of the grinding wheel to grip the die, and it is rotated at 2800 RPM.

The high carbon needle is used to confer the desired die angle, and a wooden needle is utilized to polish the die surface. The needle is smeared with the paste of boron carbide 40 and then used for polishing the die. The chuck holds the die and is rotated while the polishing needle is inserted inside the die. The skilled operator performs the HP operation. After completing the HP operation, the diameter measurement was performed with a small hole gauge, then the size of the small hole gauge was measured with the micrometer. All the observations were repeated three times, and mean values were considered for further analysis.

### 2.3. Experimental Set-Up 3: Wire Drawing Operation

**Figure 3:**
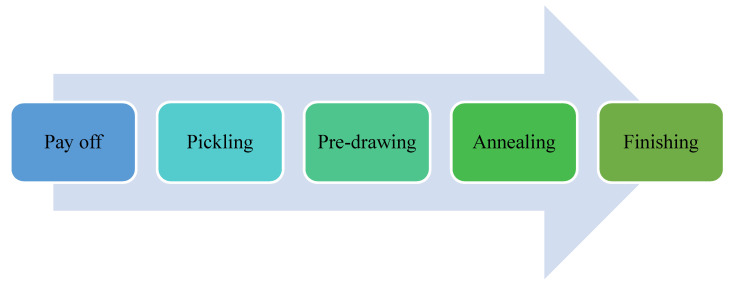
Wire drawing operation layout.

The wire drawing machine at Ball Kings Industry, Focal point, Ludhiana, Punjab, India, was used to check the performance of HPed, and AFPed WC dies. Table 3 shows the standard conditions for the continuous wire drawing set-up. The wire drawing operation consists of various processes, and the layout of the procedure is shown in Figure 3. The payoff process involves a stand onto which a wire bundle is mounted, and the wire is fed to the machines. When the wire rod’s physical impurities are removed, such as scale, oil, grease, etc., for cleaning the material for the further drawing and coating processes, this is called Pickling. The acid used for brushing was sulphuric acid maintained at 40–60 °C and 15–20% concentration.

Furthermore, the wire was immersed in a borax tank, providing a thin film on the surface of the wire, which helps protect the die and increase its life. Then, the pre-drawing and annealing were performed. The last step was finishing, and at this stage hand and AFP polished tungsten carbide dies were utilized one by one. The performance was checked at three stages of the wire drawing process. Finally, the same quantity of 5 Tons of EN9 wire material was processed or drawn from both the dies, i.e., hand and AFP polished and all set of experiments were repeated three times.

#### 2.3.1. Roughness Tester

The surface roughness tester SJ-301 (Mitutoyo, Michigan, America) with a standard detector of 10 μm, a tip radius of 5 μm (diamond stylus), and a 0.4 μm resolution with a sample length of 8 mm was utilized to assess surface roughness (Ra). It was evaluated with a differential inductance detecting technique at five different locations picked arbitrarily, and the average value was considered for further evaluation [41], with reference to a DIN EN ISO 4288 [42]. The surface roughness of abrasive flow and the hand-polished die was measured randomly from three places, and at each selected location, three readings were completed. An average of all observations were used for further analysis. In addition, a stopwatch was used to note the polishing time.

#### 2.3.2. Scanning Electron Microscopy

Surface morphological observations were conducted on the AFP and hand-polished dies by scanning electron microscope apparatus (SEM, Carl Zeiss, Bangalore, India) at an accelerating voltage of 15 kV.

## 3. Results and Discussion

### 3.1. Multi-Objective Optimization (MOO)

In the current research, the “Technique for Order Preference by Similarity to Ideal Solution” (TOPSIS) method was used to perform MOO and to select the best hand-polished die [43]. The multiple composite scores (MCS) were calculated and optimized, utilizing the “Taguchi method” to attain the optimum variables [44,45,46]. The MOO includes selecting the study’s objectives and preparing the decision matrix (TDM), as per Equation (1) [35,47]. Every row was assigned to one experiment, and every column had one response (F-Ra, % age I-Ra, TPT, etc.).

(1) $$\mathrm{TDM}=\left[\begin{array}{c} \begin{array}{ccc} e_{11} & e_{12} & \text{\_}\;\text{\_}\\ e_{21} & e_{22} & \text{\_}\;\text{\_}\\ \text{\_}\;\text{\_} & \text{\_}\;\text{\_} & \text{\_}\;\text{\_} \end{array}\\ \begin{array}{ccc} e_{i1} & e_{i2} & \text{\_}\;\text{\_}\\ \text{\_}\;\text{\_} & \text{\_}\;\text{\_} & \text{\_}\;\text{\_}\\ e_{n1} & e_{n2} & \text{\_}\;\text{\_} \end{array} \end{array}\begin{array}{c} \begin{array}{ccc} e_{1j} & \text{\_}\;\text{\_} & e_{1m}\\ e_{2j} & \text{\_}\;\text{\_} & e_{2m}\\ \text{\_}\;\text{\_} & \text{\_}\;\text{\_} & \text{\_}\;\text{\_} \end{array}\\ \begin{array}{ccc} e_{\mathrm{ij}} & \text{\_}\;\text{\_} & e_{\mathrm{im}}\\ \text{\_}\;\text{\_} & \text{\_}\;\text{\_} & \text{\_}\;\text{\_}\\ e_{\mathrm{nj}} & \text{\_}\;\text{\_} & e_{\mathrm{nm}} \end{array} \end{array}\right]$$

Normalization of decision matrix (NDM_ij_) was completed by Equation (2) and the weighted normalized matrix (WH_ij_) by Equation (3) [35,47]. In the current study, the numbers of responses in the AFP were three (3); therefore, the weight (w_j_) assigned to each individual response was 33.33%.

(2) $${\mathrm{NDM}}_{\mathrm{ij}}=\left[\frac{e_{\mathrm{ij}}}{{\left[\sum_{i=1}^{n}e_{\mathrm{ij}}^{2}\right]}^{\frac{1}{2}}}\right]\;\;\;(\mathrm{for}\;j=1,\;2,\;\ldots m)$$

(3) $${\mathrm{WH}}_{\mathrm{ij}}=\left[w_{j}{\;\times \;\mathrm{NDM}}_{\mathrm{ij}}\right]$$

The ideal best (H^+^) and ideal worst (H^−^) solutions were attained by Equation (4) and Equation (5), respectively [35,47,48,49]. H^+^ and H^−^ solutions were the utmost and least value among all response values, respectively:

(4) $$H_{j}^{+}{=\{\mathrm{best}\;(H}_{\mathrm{ij}}{)\}}_{i=1}^{n}\;H^{+}=\left\{H_{1}^{+}{,\;H}_{2}^{+}{,\;\ldots ,\;H}_{j}^{+}{,\;\ldots H}_{m}^{+}\right\}$$

(5) $$H_{j^{\prime }}^{-}{\;=\;\{\mathrm{worst}\;(H}_{{\mathrm{ij}}^{\prime }}{)\}}_{i=1}^{n}\;H^{-}=\left\{H_{1}^{-}{,\;H}_{2}^{-}{,\;\ldots ,\;H}_{j^{\prime }}^{-}{,\;\ldots H}_{m^{\prime }}^{-}\right\}$$

where j and j′ are concerned with the beneficial (m) and non-beneficial attributes (m’), respectively, and formulate separation measures (Sep) with the help of Euclidean distance, as given in Equations (6) and (7) [35,47]. Then, the multiple composite scores (MCS) of all alternatives (experiments) were calculated by Equation (8), and further optimized by the “Taguchi method” to complete MOO [50,51], or to pick the best alternative. MCS is laid in descending order [52].

(6) $${\mathrm{Sep}}_{i}^{+}=\{\overset{m}{\underset{j=1}{\sum }}{{(H}_{\mathrm{ij}}-H_{j}^{+})}^{2}{\}}^{0.5}$$

(7) $${\mathrm{Sep}}_{i}^{-}=\{\overset{m^{\prime }}{\underset{j^{\prime }=1}{\sum }}{{(H}_{\mathrm{ij}}-H_{j^{\prime }}^{-})}^{2}{\}}^{0.5}$$

(8) $$\mathrm{MCS}=\frac{{\mathrm{Sep}}_{i}^{-}}{{\mathrm{Sep}}_{i}^{+}{+\mathrm{Sep}}_{i}^{-}}$$

### 3.2. Application of Multi-Objective Optimization to Abrasive Flow Polishing

Table 4 depicts the response decision matrix of AFP used for optimization as per Equation (1). The normalized decision matrix of AFP was calculated as per Equation (2) and is presented in Table 4. The % age I-Ra has a “the higher, the better”-type response, and the F-Ra and TPT are “the lower, the better”-type responses. The calculation was assumed to be up to four significant decimal places. The weights for responses of AFP were assumed to have equal importance, and came out to be 33% due to the three responses. The weighted, normalized AFP matrix obtained by Equation (3) is also presented in Table 4. The positive ideal (best) solution was estimated by Equation (4) and the negative ideal (worst) solution by Equation (5), and the obtained results for AFP were F-Ra (0.0522, 0.1626), TPT (0.0279, 0.2233) and percentage I-Ra (0.1466, 0.0637).

Equation (6) was applied to calculate a positive separation solution (Sep_i_^+^), and Equation (7) to figure the negative separation solution (Sep_i_^−^) of the AFP. The evaluated responses are presented in Table 5. In addition, the multiple composite scores MCS were calculated by Equation (8), and are also shown in Table 5, along with their S/N ratios.

**Figure 4:**
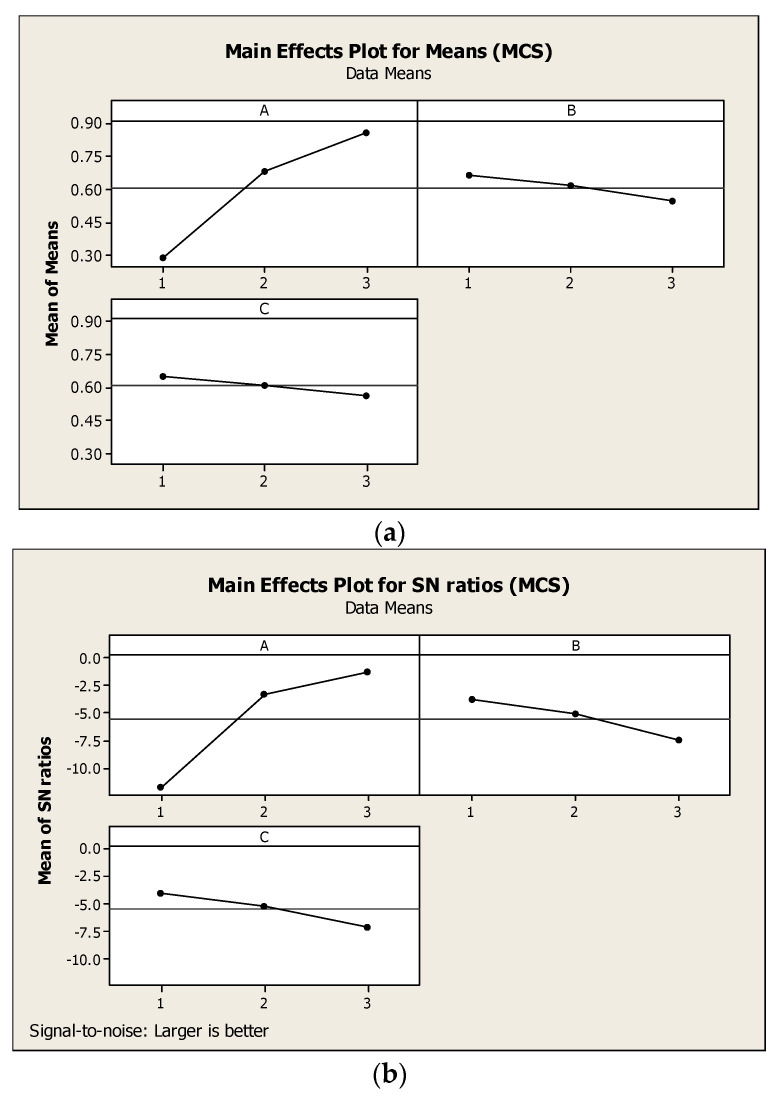
Main effects plot (**a**) means of MCS and (**b**) S/N ratios of MCS.

The main parameter effects for means and S/N ratio calculations of AFP MCS are shown in Figure 4. The MCS increased with a rise in the extrusion pressure, and decreased with a decrease in the number of cycles and abrasive particle concentration. Since MCS are a “the larger the better” attribute, from the plots, for the means and S/N ratios in Figure 4, the AFP variables at most favorable MCS are at a higher level of A, lower level of B, and lower level of C. Table 6 and Table 7 show the optimized AFP parameters for the MOO process and ANOVA, respectively.

### 3.3. Application of TOPSIS Technique to Select the Best Hand-Polished Die

The best HPed Die was chosen with the TOPSIS technique. Three-hand polishing skilled operators (HP-SO) with different die polishing experiences were considered to polish carbide dies from the industry’s available experienced operators. Hand die polishing time depended upon the operator’s skill; the skilled operator himself decided the required surface finish of the die and therefore the polishing time depended on this. So, the polishing time was not controllable and was different even when the die was polished by the skilled operator repeatedly. The observations of HP die are tabulated in Table 8 in terms of final surface roughness (HP-F-Ra) in µm, hand polishing time (HP-T) in minutes, percentage improvement in surface roughness of HP die (HP-percentage I-Ra), and HP-SO experience in years. Table 8 is also termed as the decision matrix of hand polishing responses as per Equation (1). The percentage HP-I-Ra is a “the higher, the better”-type response, and the HP-F-Ra and HP-T are “the lower, the better”-type responses. The normalized decision matrix of HP responses “NDM_ij_” was calculated as per Equation (2) and is presented in Table 8. The weights for HP responses were estimated equally, i.e., 33.33%. The weighted, normalized matrix (WH_ij_) of HP, obtained by Equation (3), is presented in Table 8. The positive ideal (best) solution was estimated by Equation (4), and the negative ideal (worst) solution by Equation (5), and the obtained results of HP were HP-F-Ra (0.0744, 0.1414), HP-T (0.0849, 0.1289) and HP-Percentage I-Ra (0.1346, 0.951).

Equation (6) was applied to calculate a positive separation solution (Sep_i_^+^) and Equation (7) to figure the negative separation solution (Sep_i_^−^). The evaluated responses of HP are presented in Table 9. The MCS of the HPed die was calculated by Equation (8), also shown in Table 9. The TOPSIS rankings obtained for each experiment of hand-polished die, along with MCS values, are presented in Table 9. Experiment 2 transpired to be the first choice of hand-polished die, followed by Experiment 1.

## 4. Comparative Analysis and Confirmation of Results

The results achieved with the Taguchi-TOPSIS method while utilizing the Equal weight technique were validated with Taguchi-GRA (Grey Relational Analysis) methodology. The optimum parameter achieved by both the methods, i.e., Taguchi-TOPSIS and Taguchi-GRA, was similar. Furthermore, confirmation experiments were performed for the MOO optimal parameters, and mean values were calculated by repeating experiments three times for further analysis. Finally, the Minitab 16 software was used to predict the MCS values. The relative error was used to check the significant difference between experimental and predicted data, as shown in Equation (9). The calculated relative error percentage for MCS is shown in Table 10. The result reveals that the predicted and experimental data were close to each other.

(9) $$\mathrm{Relative}\;\mathrm{Error}\;\mathrm{Percentage}=\left|\frac{E_{i}-P_{i}}{E_{i}}\right|\times 100$$

where E_i_ is an experimental value, and P_i_ is the predicted value.

### 4.1. Comparison between Hand and Abrasive Flow Polished Tungsten Carbide Die

Furthermore, the best-picked, hand-polished die with the TOPSIS method was compared with the AFP polished die at optimum MOO variables. The percentage changes in AFP responses achieved with MOO are shown in Table 11. The findings show a noteworthy improvement in responses with AFP polished dies compared to HPed die. The ranks of responses from I to III indicate the order of growth achieved with the MOO. Kenda et al. [53] compared AFP and hand polishing of a hardened steel surface produced by EDM and reported that hand polishing needed twice the time as AFP, with inferior surface quality.

### 4.2. Performance of Hand and Abrasive Flow Polished Die in Three-Stage Wire Drawing

The performance of the AFP and the hand-polished die was evaluated in the three-stage wire drawing operation. The HP-SO 1 was selected per the TOPSIS rank (refer to Table 12) to polish three carbide dies. The die’s size was picked as per the size mentioned in Table 3, i.e., standard multi-stage wire drawing operation at Ball Kings, Ludhiana, India. In this research, the dies’ performances were evaluated at the third, fourth, and fifth stages of the standard wire drawing operation. In addition, the AFP die polishing parameters were considered (refer to Table 6), obtained with the MOO of the AFP process. The surface deterioration rate may depend upon the initial roughness. However, in the present work, it was ensured that hand-polished and abrasive flow polished dies had almost equal initial surface roughness so that the effect of wire drawing could be noticed without any bias.

As shown in Table 12, at the first stage of wire drawing, the percentage of deterioration of Ra with hand-polished die was 38.93, and with the abrasive flow polished die this was 26.94. So, there was 11.93% more surface decline of hand-polished dies than abrasive flow polished die. In the second stage of wire drawing, the percentage deterioration in surface roughness with HP die was 50.30, and with AFPed die this was 42.97; therefore, there was 7.33% more surface decline of HPed die compared with AFPed die. Finally, in the third stage of wire drawing, the percentage deterioration in Ra with HPed die was 60.71%, and with AFPed die this was 51.50. So, there was 9.21% more surface decline of HPed dies as compared with AFPed die. Accordingly, at each stage, i.e., first, second and third, the bearing diameter of the HPed die enlarged by 25% compared with the AFPed die.

**Figure 5:**
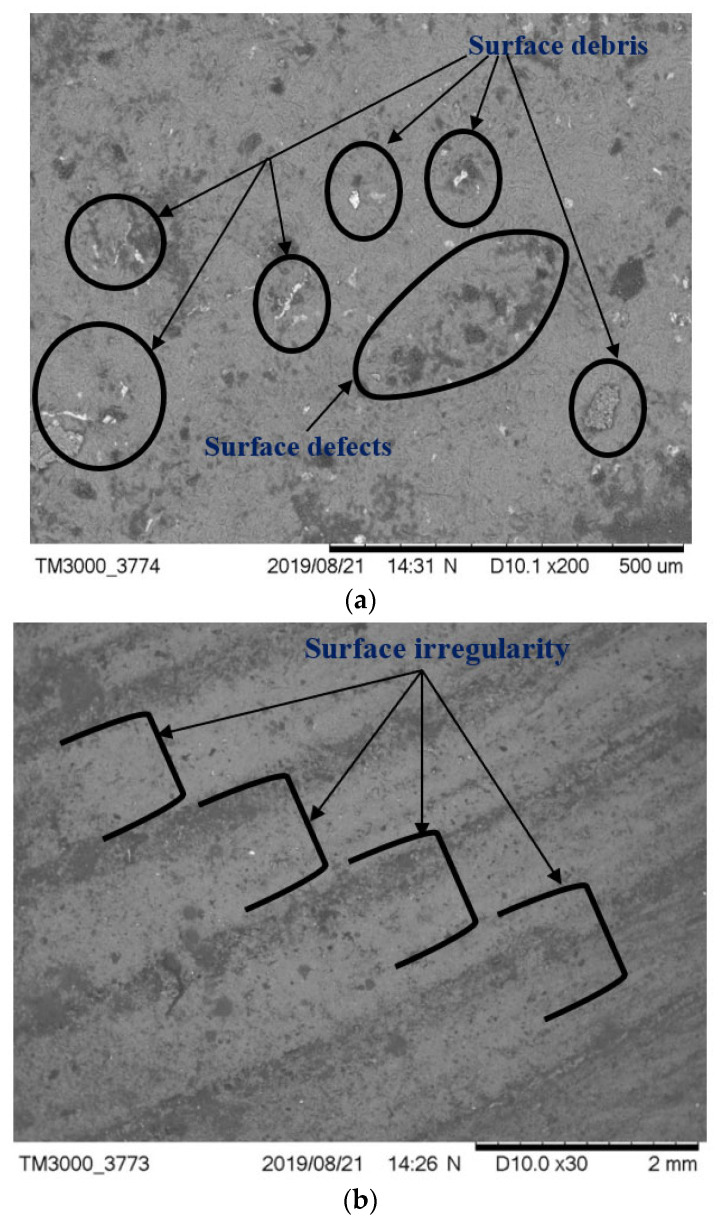
SEM micrograph of (**a**) abrasive flow polished die (**b**) hand polished die.

There was a reduced amount of wear and tear of the abrasive flow polished die compared with the hand-polished die. The magnified view of SEM of the abrasive flow polished die in Figure 5a shows that all surfaces were polished uniformly; the polished surface was parallel to the wire to be drawn. Figure 5a also indicates the surface debris and surface defects. In the hand polishing of die, although the surface looked good, the surface deterioration was greater than that of abrasive flow polished die. Much of the surface is folded metal and not parent metal, so the drawing wire pulls out the folded metal after a short period of use, or dislodges the upper surface of the die [2]. This results in several defects due to the poor surface finish of the die, such as U-shaped cracks, pitting, wear rings, and vertical and horizontal cracks. The wire continually contacts the die hole during the drawing process, releasing the tungsten carbide particles and generating a rough surface. The rough surface scrapes metal bits off the surface of the steel wire, weakening the lubrication and increasing the wear rate [3,4].

Figure 5b also indicates that the surface is not very uniform. There is no particular lay to the finish. Therefore, some areas could cause more friction than others, possibly resulting in uneven wire flow and further deterioration occurring. Other reasons may be that when the wire initially touches the dies, the tungsten carbide grains are torn out, scratching the die surface. If the surface is rough, there are more chances of tearing out of grains. Abrasive wear results in the bearing and cone area of the die, and this wear may be due to lubricant impurities and the presence of oxide elements on the wire [54].

### 4.3. Contour Plots of Abrasive Flow Polished Die

**Figure 6:**
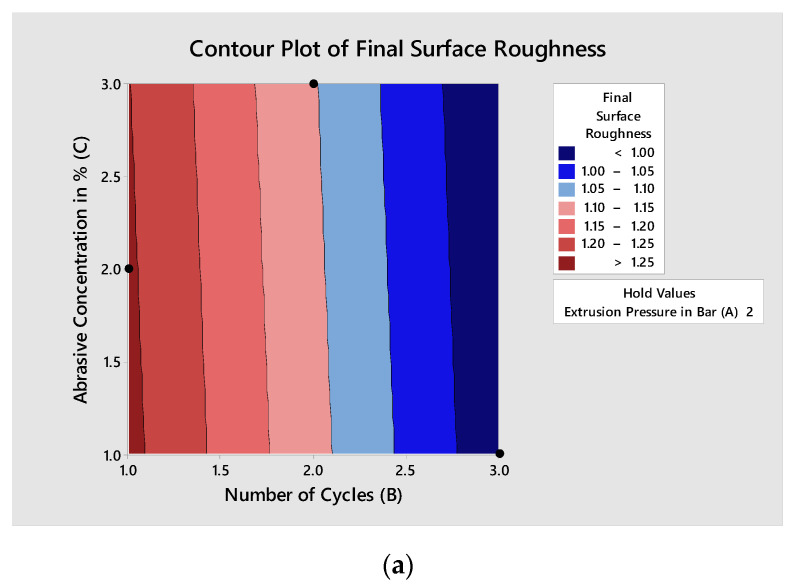

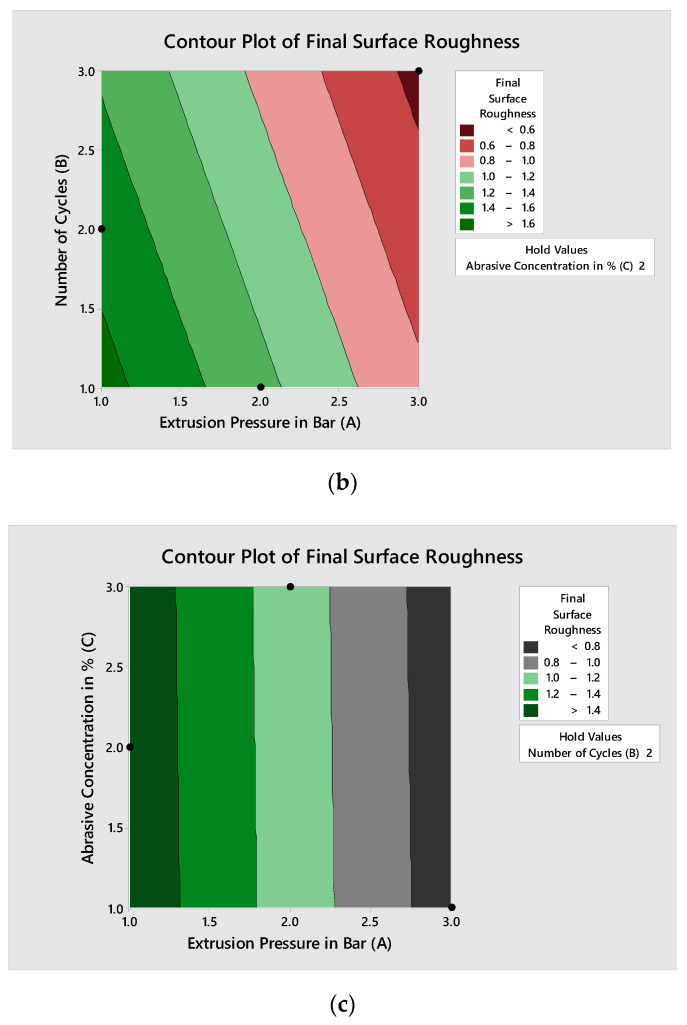
Contour plots for final surface roughness: (**a**) plot between abrasive concentration and the number of cycles; (**b**) plot between the number of cycles and extrusion pressure; (**c**) plot between abrasive concentration and extrusion pressure.

**Figure 7:**
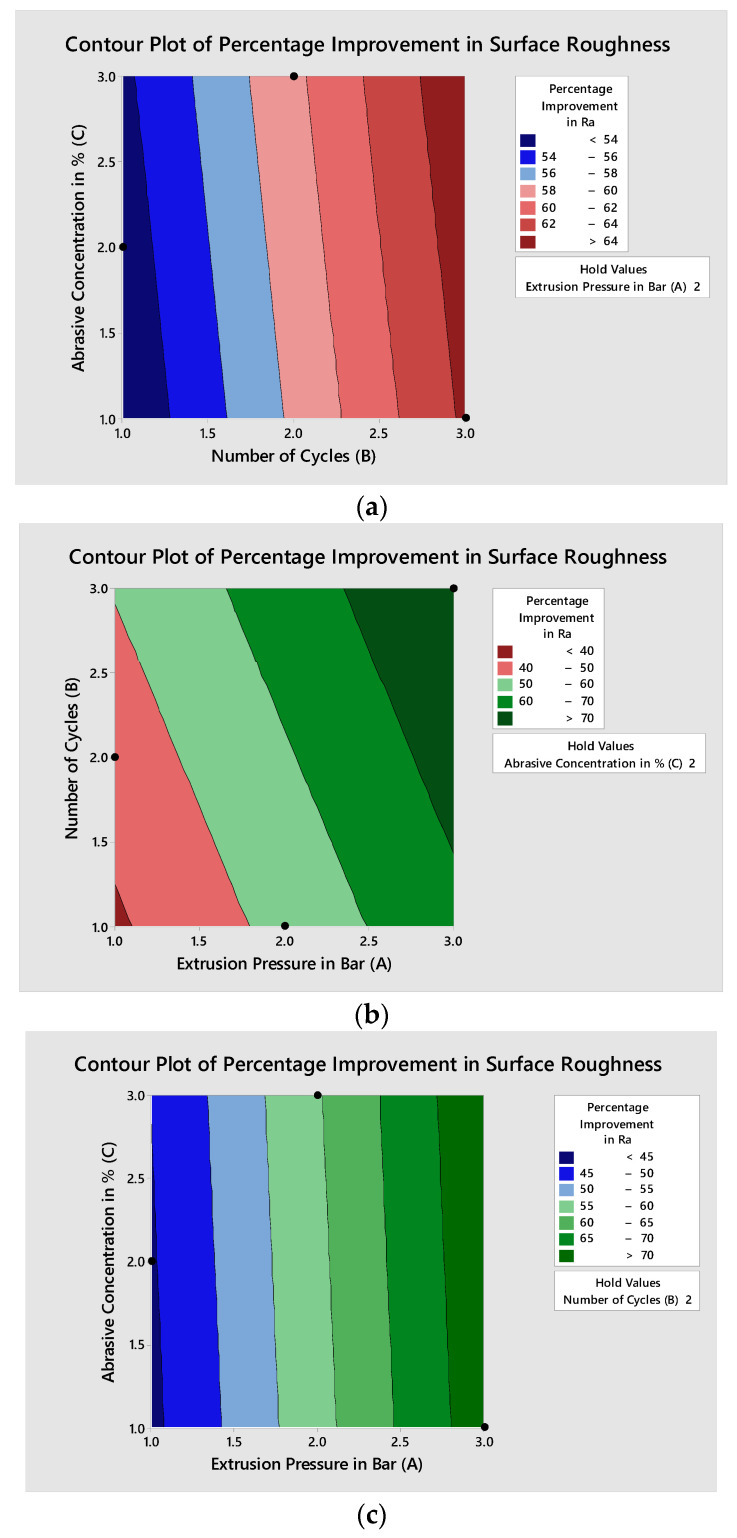
Contour plots for percentage improvement in surface roughness: (**a**) plot between abrasive concentration and the number of cycles; (**b**) plot between the number of cycles and extrusion pressure; (**c**) plot between abrasive concentration and extrusion pressure.

**Figure 8:**
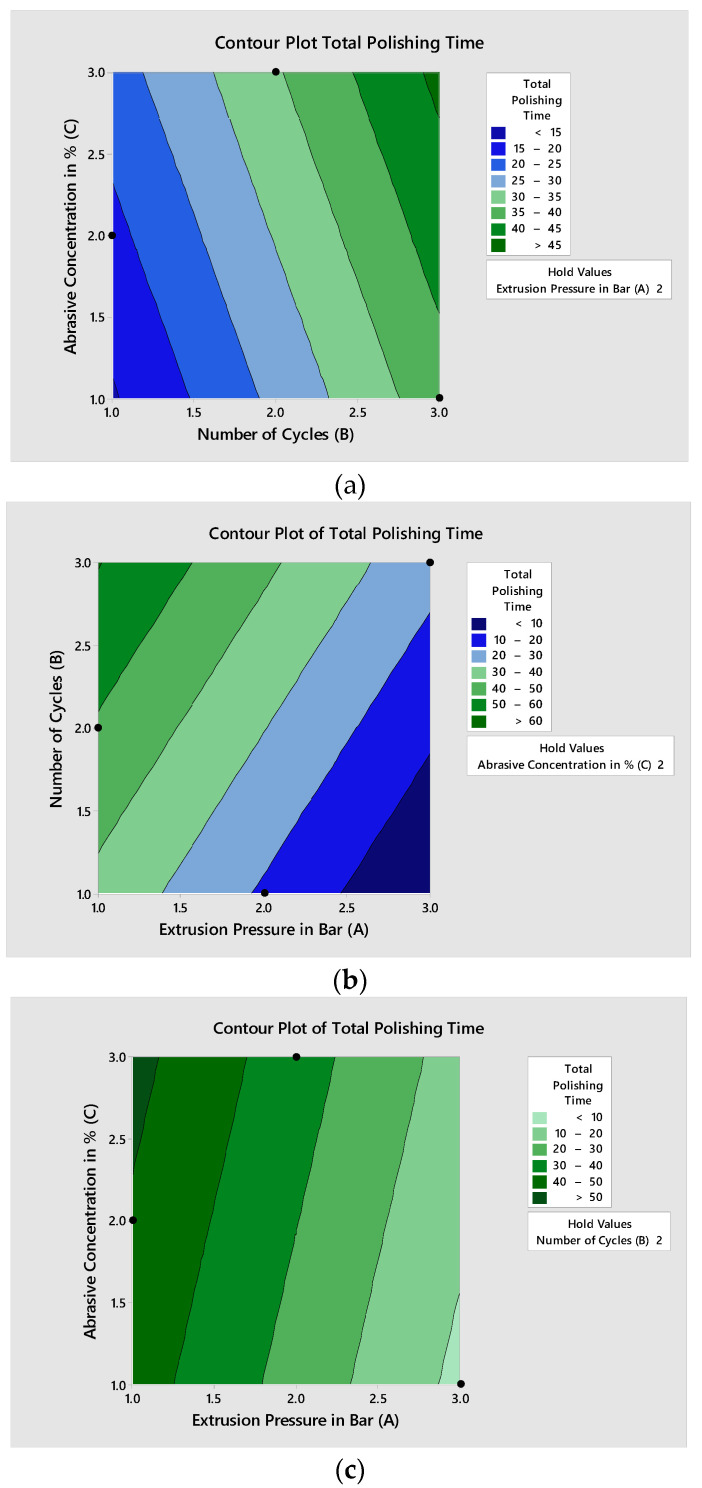
Contour plots for total polishing time: (**a**) plot between abrasive concentration and a number of cycles; (**b**) plot between the number of cycles and extrusion pressure; (**c**) plot between abrasive concentration and extrusion pressure.

The effects of AFP variables’ extrusion pressure (A), the number of cycles (B), and abrasive concentration (C) on the individual responses’ final surface roughness, percentage improvement in surface roughness, and total polishing time can be seen from the contour plots in Figure 6, Figure 7 and Figure 8, respectively. Minitab 16 software was utilized to draw contour plots.

In Figure 6a, the minimum value of the response final Ra is characterized by a dark red color. The contour plots are drawn for the linear model of the response at a 95% confidence level. The contour line encircling this area gives the value of Ra as less than 1 µm. The number of cycles (B) are plotted on x-axes and abrasive concentration (C) on the y-axes. In Figure 6b, the minimum value of the response final Ra is characterized by a dark red color. The extrusion pressure (A) is plotted on x-axes and the number of cycles (B) are plotted on the y-axes. The Ra lies around 0.603 to 0.609 µm, and B*A varies between levels (2.862, 2.962) to (2.268, 2271). Thus, the extrusion pressure has a substantial role in reducing final Ra, and has a significant effect. For the rest of the contour graphs, the area representing the minimum final Ra is negligible, as in Figure 6c. Therefore, their explanation was not considered, and they had no significant effect on C*A and C*B.

Similarly, the contour plot for percentage improvement in Ra is shown in Figure 7, and the dark red color indicates the maximum percentage improvement in the Ra area where optimal variable blend lies. The dark blue color can be seen in the contour plot between B*A (2.365, 2.386) and (2.298, 1.486), with a percentage improvement in Ra from 70 to 70.11 (refer to Figure 7b). For the C*A (2.724, 2.793) to (2.81, 1.02) with percentage improvement in Ra of more than 70%, refer to Figure 7b. In Figure 7c, for C*B, the contour area without the dark blue color shows a non-significant effect on the percentage improvement in Ra.

Similarly, the contour plot for total polishing time is shown in Figure 7, and the dark blue color indicates the minimum total polishing time area where the optimal variable blend lies, and the TPT value is less than 10. The dark blue color can be seen in the contour plot between B*A (2.988, 1798) and (2.471, 1.021), with TPT less than 10 min. The C*A was (2.995, 1.501) to (2.866, 1.121). For C*B, the contour area without the dark blue color shows a non-significant effect on TPT. The coded variables and their actual values can be seen in Table 1. The extrusion pressure varies from 65 Bar to 105 Bar, the number of cycles from 80 to 180, and abrasive particle concentration from 50% to 60%.

As the number of cycles increases, the percentage of improvement in the surface finish also increases. With an increase in the number of cycles, the number of times the abrasives come into contact with the workpiece increases, so the percentage improvement in surface finish increases, and there is a greater percentage improvement in the surface finish in the initial working cycles—after that, the percentage improvement in the surface finish was lesser. This is because a major part of the initial roughness of the workpiece is removed in a few cycles at the beginning of AFM [16]. It is evident that as the pressure increases, the percentage improvement in the surface finish also increases. When the pressure increases, the normal force acting on each grain increases, resulting in a deeper indentation on the workpiece surface. If the axial force on an abrasive is more than the resistance offered by the workpiece material, removing the peaks over the surface of the workpiece takes place, leading to an improved surface finish [14]. At higher pressure, fewer cycles are required to attain a particular surface finish value than low pressure. At low pressure, the indentation force exerted on the workpiece surface is low, which results in a low finishing rate. At high extrusion pressure, the time required to finish a component is reduced because the medium provides greater and deeper indentations on the surface of the workpiece. As the number of cycles increases, the polishing time also increases. With increased extrusion pressure, media flow speed increases and polishing time decreases [28]. With an increase in extrusion pressure, polishing time decreases.

In the present research, the effect of three abrasive flow polishing parameters was studied in terms of final surface roughness, polishing time, and improvement in surface finish. Other abrasive flow polishing parameters, such as type of media and media flow rate, can be considered for future research as the parameters decided in the present case were as per machine set-up constraints. In the present case, abrasive flow polished die performance was compared with hand polishing, and is taken as a case study. Still, there are more polishing techniques utilized in die polishing, such as robotic polishing [10], ultrasonic polishing [55], and computer-controlled polishing processes [56]. Still, some advanced variants of abrasive flow machining have been developed, and work can further be analyzed, such as magneto abrasive flow machining [57], and the recent trends in abrasive flow machining presented in [58].

## 5. Conclusions

In the present research work, polishing of tungsten carbide (WC) wire drawing die has been explored with abrasive flow polishing (AFP) to compare its performance with conventional hand polishing (HP). The Taguchi-TOPSIS Equal-Weight technique was used to achieve multi-objective optimization of final surface roughness (F-Ra), percentage improvement of surface roughness (percentage I-Ra), and total polishing time (TPT). Additionally, the TOPSIS approach was used to identify the best hand-polished die. The applied abrasive flow polishing technique uniquely contributes to the die polishing industry, where dependence on skilled operators can be reduced with the proposed methodology. Furthermore, the proposed method applied to the die polishing process, while considering multiple responses for hand and abrasive flow polishing, has a unique advantage as it can reduce the polishing time and enhance the surface quality of the wire drawing die. As a result, it also achieved better results when the performance of the abrasive flow polished die was checked in a multi-stage wire drawing operation. Based on experimental observations and statistical analysis of the results, the following conclusions have been drawn:

- AFPed and HPed die performance in multi-stage wire drawing operation revealed that abrasive flow processing provides better surface quality than hand polishing in terms of wear rate. There were 11.93%, 7.33%, and 9.21% lower wear and tear of AFP surfaces than hand-polished surfaces, at the first, second and third stages of wire drawing operation;
- The bearing diameter of HPed dies enlarged by 25% more than the AFPed dies. As a result, the AFP offered better surface quality (Ra) in contrast to hand polishing. AFP can reduce the dependency on expensive and increasingly difficult-to-find die finishers or skilled operators. In addition, the AFP polishes all surfaces uniformly within a reasonable amount of time, i.e., a percentage time saving of 87.50;
- It was found from the means, S/N plots, and ANOVA analysis (at 95% confidence level) of AFP that the extrusion pressure had the maximum significance on the MCS calculations with a contribution of 91.21%. In contrast, abrasive particle concentration was seen to be influenced significantly less;
- The multi-objective optimization was performed with the technique of the Taguchi-TOPSIS-Equal-Weight. The AFP results were: polishing parameters at extrusion pressure of 105 bars, number of cycles, 80, and an abrasive particle concentration of 50%. There was an improvement of 87.50% in TPT, 60.68% in F-Ra, and 27.06% in percentage I-Ra compared with the best hand-polished die selected with the TOPSIS method;
- The results of the TOPSIS method to pick the best hand-polished die revealed that the skilled operator, having five years of experience, came out to be the first choice, having HP-F-Ra 2.256 μm, HP-T of 32 min., and HP-% age I-Ra of 51.25. This was followed by the same skilled operator, having HP-F-Ra 2.167 μm, HP-T of 28 min., and HP-% age I-Ra of 48.89.

## Figures and Tables

**Table 1:** Abrasive flow polishing variables.

Sr. No.	Variables	Levels		
		1	2	3
A	Extrusion Pressure (Ep), Bar	65	85	105
B	Number of Cycles (Noc)	80	130	180
C	Abrasive Concentration (Ac), Percentage	50	55	60

**Table 2:** Taguchi L9 orthogonal array, experimental observations.

Exp. No.	Extrusion Pressure (Ep), Bar	Number of Cycles (Noc)	Abrasive Concentration (Ac), %	I-Ra (µm)	F-Ra (µm)	Percentage I-Ra	PT One Cycle (s)	TPT (min)
1	65	80	50	2.612	1.708	34.61	24	32
2	65	130	55	2.834	1.498	47.14	24	52
3	65	180	60	2.792	1.439	48.46	24	72
4	85	80	55	2.871	1.231	57.12	11	15
5	85	130	60	2.549	1.019	60.02	11	24
6	85	180	50	2.783	0.942	66.15	11	33
7	105	80	60	2.634	0.879	66.63	7	9
8	105	130	50	2.456	0.721	70.64	7	15
9	105	180	55	2.694	0.549	79.62	7	21

I-Ra: initial surface roughness, F-Ra: final surface roughness, % age I-Ra: percentage improvement of surface roughness, PT: polishing time, TPT: total polishing time.

**Table 3:** Standard multi-stage wire drawing operation.

No. of Stages	1st	2nd	3rd	4th	5th	6th	7th
The diameter of the drum (mm)	600	595	590	590	585	585	585
Finishing speed (RPM)	21	24	33	42	56	61	70
Die material	Tungsten Carbide						
Material to be drawn	EN9						
Inlet wire size (mm)	5.5	5.05	4.63	4.23	3.85	3.5	3.2
Finished wire size (mm)	5.05	4.63	4.23	3.85	3.5	3.2	2.92
% age reduction	8.18	8.31	8.63	8.9	9.09	8.5	8.75

**Table 4:** Decision matrix of abrasive flow polishing responses.

Exp. No.	Decision Matrix			Normalized Decision Matrix			Weighted, Normalized Matrix		
	F-Ra	TPT	% Age I-Ra	F-Ra	TPT	% Age I-Ra	F-Ra	TPT	% Age I-Ra
1	1.708	32	34.61	0.4877	0.2978	0.1912	0.1626	0.0993	0.0637
2	1.498	52	47.14	0.4277	0.4839	0.2604	0.1426	0.1613	0.0868
3	1.439	72	48.46	0.4109	0.6700	0.2677	0.1370	0.2233	0.0892
4	1.231	15	57.12	0.3515	0.1396	0.3155	0.1172	0.0465	0.1052
5	1.019	24	60.02	0.2909	0.2233	0.3315	0.0970	0.0744	0.1105
6	0.942	33	66.15	0.2690	0.3071	0.3654	0.0897	0.1024	0.1218
7	0.879	9	66.63	0.2510	0.0837	0.3680	0.0837	0.0279	0.1227
8	0.721	15	70.64	0.2059	0.1396	0.3902	0.0686	0.0465	0.1301
9	0.549	21	79.62	0.1567	0.1954	0.4398	0.0522	0.0651	0.1466

**Table 5:** Separation measures, MCS and S/N ratios of abrasive flow polishing.

Exp. No.	Sep_i_^+^	Sep_i_^−^	MCS	S/N Ratio
1	0.1553	0.1241	0.4441	−7.051
2	0.1718	0.0652	0.2750	−11.213
3	0.2206	0.0361	0.1408	−17.030
4	0.0792	0.1872	0.7026	−3.065
5	0.0739	0.1693	0.6960	−3.148
6	0.0869	0.1527	0.6373	−3.914
7	0.0395	0.2188	0.8472	−1.441
8	0.0298	0.2109	0.8762	−1.148
9	0.0372	0.2099	0.8494	−1.418

**Table 6:** Abrasive flow polishing parameters at optimum MCS and response value.

Optimum AFP (Factor/Level)		Optimum MCS		Responses	Mean	S/N Ratio
		Mean	S/N Ratio			
Ep (A3)	105 Bar	0.9595	−0.3591	F-Ra (µm)	0.887	1.0415
Noc (B1)	80			Percentage I-Ra	65.12	36.2743
Ac (C1)	50			TPT (min)	4	−12.0412

**Table 7:** ANOVA results in MCS.

Resource	Degree of Freedom	Sum of Square	Variance	Fisher’s Value	Probability	Contribution (%)
**MCS means**						
Ep	2	0.51172	0.255859	36.18	0.027	91.21
Noc	2	0.02268	0.01134	1.6	0.384	4.04
Ac	2	0.01249	0.006245	0.88	0.531	2.23
Residual Error	2	0.01414	0.007072			2.52
Total	8	0.56103				100.00
**MCS S/N ratios**						
Ep	2	183.31	91.656	11.87	0.078	78.32
Noc	2	19.93	9.963	1.29	0.437	8.52
Ac	2	15.37	7.683	0.99	0.501	6.57
Residual Error	2	15.45	7.725			6.60
Total	8	234.05				100.00

**Table 8:** Observations and decision matrix of HP responses.

Exp. No.	HP-SO	Decision Matrix			Normalized Matrix			Weighted, Normalized Matrix		
		HP-F-Ra (μm)	HP-T (min)	HP-% Age I-Ra	HP-F-Ra	HP-T	HP-% Age I-Ra	HP-F-Ra	HP-T	HP-% Age I-Ra
1	1	2.167	29	48.89	0.2231	0.2548	0.3405	0.0744	0.0849	0.1135
2	1	2.256	32	51.25	0.2322	0.2811	0.3569	0.0774	0.0937	0.1190
3	1	2.743	35	57.98	0.2824	0.3075	0.4038	0.0941	0.1025	0.1346
4	2	2.998	38	48.01	0.3086	0.3339	0.3344	0.1029	0.1113	0.1115
5	2	3.127	40	44.93	0.3219	0.3514	0.3129	0.1073	0.1171	0.1043
6	2	3.258	39	49.25	0.3354	0.3426	0.3430	0.1118	0.1142	0.1143
7	3	3.878	42	40.98	0.3992	0.3690	0.2854	0.1331	0.1230	0.0951
8	3	3.989	40	42.58	0.4106	0.3514	0.2966	0.1369	0.1171	0.0989
9	3	4.091	44	44.68	0.4211	0.3866	0.3112	0.1404	0.1289	0.1037

**Table 9:** Separation measures and MCS of hand-polished die and TOPSIS ranks.

Exp. No.	Sep_i_^+^	Sep_i_^−^	MCS	Rank
1	0.0211	0.0814	0.7941	2
2	0.0156	0.0759	0.8294	1
3	0.0264	0.0663	0.7148	3
4	0.0452	0.0445	0.4962	4
5	0.0551	0.0363	0.3968	6
6	0.0517	0.0374	0.4200	5
7	0.0803	0.0094	0.1044	8
8	0.0789	0.0128	0.1394	7
9	0.0851	0.0086	0.0917	9

**Table 10:** Predicted and experimental abrasive flow polished MCS at optimal parameters.

Response	Factor/Level	Predicted	Experimental	Relative Error (%)
MCS	A3, B1, C1	0.9595	0.9188	4.43

**Table 11:** Percentage change in abrasive flow polishing responses in comparison to hand polishing.

Responses	HPed Die	AFPed Die	Percentage Change in AFP Polished Die
	Response Value		
F-Ra (µm)	2.256	0.887	−60.68 ^II^
% age I-Ra	51.25	65.12	27.06 ^III^
TPT (min)	32	4	−87.50 ^I^

^I, II, and III^ are ranks of AFP responses as per improvement; negative sign means improvement for F-Ra and TPT; positive sign means improvement for percentage I-Ra.

**Table 12:** Observations of three-stage wire drawing operation.

Polishing Method	Number of Stages	Surface Roughness, Ra (μm)		Percentage Reduction in Ra	Bearing Diameter of Die (mm)		Increase in Bearing Diameter of Die (mm)
		Before Drawing	After Drawing		Before Drawing	After Drawing	
Hand polished	First	2.638	3.665	38.93	4.63	4.66	0.03
	Second	2.187	3.287	50.30	4.23	4.28	0.05
	Third	2.273	3.653	60.71	3.85	3.89	0.04
AFP polished	First	0.798	1.013	26.94	4.63	4.65	0.02
	Second	0.854	1.221	42.97	4.23	4.27	0.04
	Third	0.831	1.259	51.50	3.85	3.89	0.03

## Data Availability

Not applicable.
